# Protein S deficiency: Recurrent ischemic stroke in young

**DOI:** 10.4103/0972-2327.56319

**Published:** 2009

**Authors:** Amit Hooda, P. D. Khandelwal, Puneet Saxena

**Affiliations:** Department of Medicine, SMS Medical College and Hospital, Jaipur, Rajasthan, India

**Keywords:** Protein S deficiency, recurrent ischemic stroke, young stroke

## Abstract

Stroke in young poses a major health problem. Thrombophilic factors have been implicated in 4-8% of the young strokes worldwide. Protein S deficiency is a rare cause of recurrent ischemic stroke in young population. Only a few sporadic cases have been described in the literature. We are reporting a case of protein S deficiency-related recurrent ischemic stroke in a 16-year-old girl. Early diagnosis and targeted approach can help such patients to prevent recurrent thrombotic episodes.

## Introduction

Protein S is a naturally occurring vitamin K-dependent protein, which in conjunction with active protein C, inhibits the clotting cascade. Protein S deficiency is known to be of clinical significance in patients with deep venous thrombosis or pulmonary emboli. The overall estimated incidence of deep vein thrombosisis is one episode for every 1,000 persons. Protein S deficiency has been also found to be associated with cerebrovascular occlusion, although the exact role is controversial.

## Case Report

A 16-year-old girl presented with acute onset left sided hemiparesis without loss of consciousness. General physical examination was unremarkable. Neurological examination revealed findings consistent with left-sided hemiparesis. A similar episode occurred three years back. No precipitating factors such as chronic drug intake were present. Family history was negative for vascular events or other predisposing factors for stroke.

**Figure 1:**
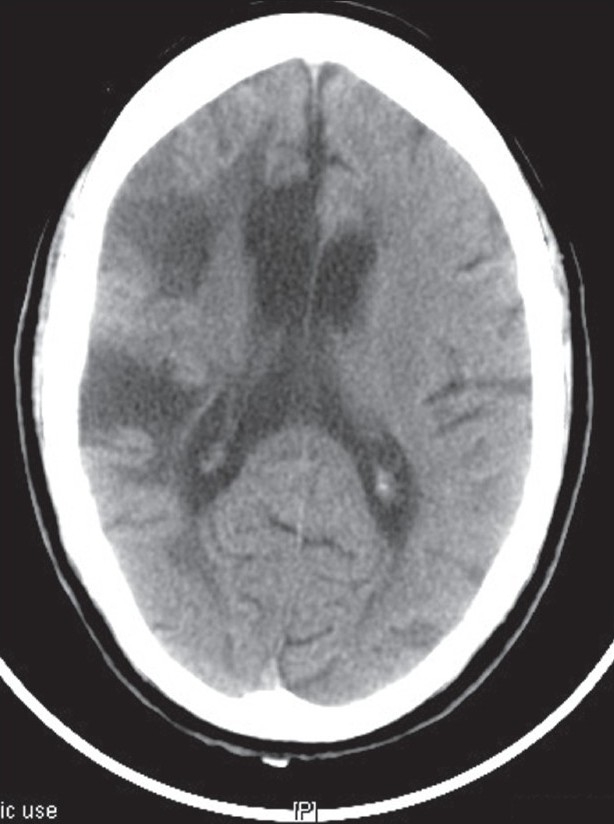
CT head revealed a wedge shaped acute infarct in right middle cerebral artery territory along with areas of enchephalomalacia and gliosis in right fronto-temporal and temporo-parietal lobe and in paraventricular white matter with ex-vacuo-dilation of frontal horn of lateral ventricle, suggestive of chronic infarct of middle cerebral artery territory

CT head [Figure 1] revealed a wedge shaped acute infarct in right middle cerebral artery territory alongwith areas of enchephalomalacia and gliosis in right fronto-temporal and temporo-parietal lobe and in paraventricular white matter with ex-vacuo-dilation of frontal horn of lateral ventricle, suggestive of chronic infarct of middle cerebral artery territory. Magnetic resonance angiography (MRA) showed narrow lumen and caliber of right middle cerebral artery alongwith absent flow in right supraclinoid internal carotid artery. Routine hematological examination along with lipid profile, coagulation profile, echocardiography, and duplex scanning were unremarkable. Vasculitis profile was negative. Cerebrospinal fluid examination did not reveal any abnormality.

Workup for thrombophilias revealed reduced protein S function (15% of normal) alongwith protein C; whereas, antithrombin III, anticardiolipin antibodies, and lupus anticoagulant were within normal limits. A diagnosis of protein S deficiency was kept and the patient was managed with intravenous heparin followed by oral anticoagulants. Neurological functions improved and patient was discharged on oral anticoagulants. Repeat thrombophilic profile after three months revealed protein S functional activity 42% of the normal with patient showing remarkable recovery.

## Discussion

Stroke in young population has a high incidence of approximately 24–35%, according to some studies in India. Abraham *et al*.[1] from Vellore reported an incidence of 25% in population less than 40 years of age. Munts *et al*.[2] reported that idiopathic coagulation disorders were found in about a quarter of young stroke patients, although the clear-cut data has been lacking from India. Carod-A *et al*.[3] studied about ischemic stroke subtypes and prevalence of thrombophilia in Brazilian stroke patients. They examined 130 consecutive young and 200 elderly patients. Prevalence of thrombophilia was, respectively: protein S deficiency (11.5% versus 5.5%), protein C deficiency (0.76% versus 1%). They concluded that prothrombotic conditions were more frequent in stroke of undetermined causes.

The importance of thrombophilic disorders in arterial stroke has been debatable. Ischemic stroke has been reported as a rare manifestation of protein S deficiency. Girolami *et al*.[4] and Sie *et al*.[5] were among the first who reported the association of familial deficiency of protein S as a cause of ischemic stroke in young. Wiesel *et al*.[6] studied 105 patients with protein S deficiency, out of which 14 had arterial thrombotic accidents involving the central nervous system or the myocardium, while most studies revealed a weaker association between the two.[7–9] Douay *et al*.[8] reported that hereditary deficiencies of coagulation inhibitors are rare in ischemic stroke patients under 45 years and their systematic detection seems to be of poor interest. Mayer *et al*.[9] also supported the fact that acquired deficiency of free protein S is not a major risk factor for ischemic stroke.

In this 16-year-old patient without any risk factors, the acquired factor S deficiency possibly played a role in the recurrent ischemic stroke. Factor S deficiency should be considered in venous stroke, recurrent pulmonary embolism, unusual site of venous occlusion, family history of vascular events, and stroke in young population. Aetiology of such vascular events in young must be thoroughly investigated so as to guide prevention and treatment of this devastating disease. Measurement of total and free protein S levels should be a part of the evaluation for any young adult who has had a stroke.
